# Purification and characterization of a novel immunoregulatory peptide from *Sipunculus nudus* L. protein

**DOI:** 10.1002/fsn3.3695

**Published:** 2023-09-26

**Authors:** Haisheng Lin, Wan Li, Ruikun Sun, Cheng Xu, Chaohua Zhang, Jialong Gao, Wenhong Cao, Xiaoming Qin, Saiyi Zhong, Yibin Chen

**Affiliations:** ^1^ College of Food Science and Technology Guangdong Ocean University Zhanjiang China; ^2^ Guangdong Provincial Key Laboratory of Aquatic Product Processing and Safety Guangdong Ocean University Zhanjiang China; ^3^ Guangdong Province Engineering Laboratory for Marine Biological Products Guangdong Ocean University Zhanjiang China; ^4^ Guangdong Provincial Engineering Technology Research Center of Marine Food Guangdong Ocean University Zhanjiang China; ^5^ Empress Therapeutics Cambridge Massachusetts USA; ^6^ Hainan Semnl Biotechnology Co. Ltd. Chengmai China

**Keywords:** enzymatic hydrolysis, immunoregulatory peptide, purification, RAW 264.7 macrophage, *Sipunculus nudus* L.

## Abstract

This study aimed to purify and characterize immunoregulatory peptides from *Sipunculus nudus* L. and to explore the underlying mechanisms. Ultrafiltration, gel filtration chromatography, and reverse phase high‐performance liquid chromatography (RP‐HPLC) were used to purify the peptide following enzymatic hydrolysis. Rates of lymphocyte proliferation and phagocytosis as well as nitric oxide (NO) production levels were used as indicators of immunoregulatory activity to screen the fractions. The amino acid sequence of the peptide, designated as SNLP, was identified as Arg–Val–Lys–Gly–Lys–Ile–Leu–Ala–Lys–Arg–Leu–Asn (RVKGKILAKRLN) by liquid chromatography–tandem mass spectrometry (LC–MS/MS). Treatment with the synthetic SNLP increased the proliferation and phagocytosis of RAW 264.7 macrophages and promoted the secretion of tumor necrosis factor‐ɑ (TNF‐α), interleukin‐6 (IL‐6), interleukin‐1β (IL‐1β), and NO levels. The mRNA levels of these cytokines and iNOS were also increased by SNLP. Our results provide preliminary evidence suggesting that SNLP acts as a dual immunomodulatory peptide with immunostimulatory and anti‐inflammatory activities. In summary, SNLP derived from *Sipunculus nudus* L. is a potent immunoregulatory peptide and represents a potential functional food or immunoregulatory drug.

## Graphical abstract

A novel natural potent immunoregulatory peptide: SNLP was identified from *Sipunculus nudus* L. Synthesized SNLP increased the proliferation and phagocytosis of RAW 264.7 macrophages, and promoted the secretion of TNF‐ɑ, IL‐6, IL‐1β, and NO. Our results provided preliminary evidence that SNLP is the two‐way immunomodulatory peptides with immunostimulatory and anti‐inflammatory activities. In summary, SNLP derived from *Sipunculus nudus* L. is a potent immunoregulatory peptide and it has great potentials as functional food or immunoregulatory drug.

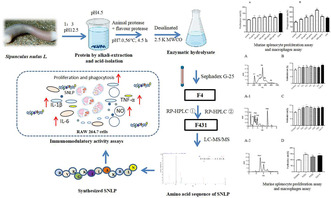

## 1 INTRODUCTION

The immune system encompasses the physiological activities of cells, lymphoid organs, cytokines, and humoral factors. A dysfunctional immune system increases the risk of infection and disease (Parkin & Cohen, 2001). These diseases include various cancers and other conditions, such as lung cancer, melanoma, Crohn's disease, and rejection after organ transplantation (Carbone et al., 2015; Xu et al., 2020). Multiple drugs have been developed to modulate the immune responses related to these diseases. Some of the drugs were designed to suppress the immune response. The drugs include infliximab (Nakashima et al., 2020), azathioprine (McNally & Carey, 2021), abatacept (Galbraith et al., 2020), and tacrolimus (Epps & Venkata, 2021). Others like ipilimumab and nivolumab block immune checkpoints to activate immune cell proliferation and response (Shitara et al., 2022). However, these drugs are limited by toxic side effects and high cost, and therefore not suitable for chronic or prophylactic use.

Bioactive peptides, composed of 2–20 amino acids, contribute to biological activity and have a positive effect on human health (Hu, Pan et al., 2022, Hu, Yang et al., 2022; Xu et al., 2020). Over the past few decades, bioactive peptides derived from natural resources, with low toxicity and cost but high degree of biological activity, have been reported to reduce or prevent chronic disease risk and provide immune protection (Shafique et al., 2023), and thus used as health and functional food to effectively modulate immune response (Cai et al., 2020; Milica et al., 2022; Ye et al., 2022).

*Sipunculus nudus* L., commonly known as sandworm, lives in sediment of the intertidal zone. Traditionally considered as a tonic and medicine for treating various diseases in the southern regions of China, this earthworm‐shaped marine worm is mainly distributed in the Atlantic Ocean, Indian Ocean, Pacific Ocean, and the coastal areas of Guangdong, Guangxi, Hainan, and Taiwan in China. Polysaccharides derived from *S. nudus* L. exhibit anti‐inflammatory and antinociceptive activities (Zhang et al., 2011), radioprotective effects (Li et al., 2016), as well as antitumor and antivirus activities (Su et al., 2016). However, the protein component, accounting for 80% of the dry weight of *S. nudus* L., has yet to be analyzed. Our preliminary study suggested that the aqueous extracts and collagen peptides derived from *S. nudus* L. possess wound‐healing activity (Lin et al., 2021; Zheng et al., 2020). Further, the enzymatic hydrolysate of *S. nudus* L. showed immunoregulatory activity and suggests that the worm is an excellent candidate for producing immunoregulatory peptides (Sun et al., 2018).

In this study, we were particularly interested in purifying the peptide with immunoregulatory activity from *S. nudus* L. and elucidate the underlying regulatory mechanisms. Enzymatic hydrolysis, ultrafiltration, and chromatographic methods coupled with liquid chromatography–tandem mass spectrometry (LC–MS/MS) were utilized to purify and identify the peptide. Immune cell proliferation, cytokine levels, and the levels of signaling molecule NO were used to demonstrate the immunoregulatory activity of the peptide. This study is expected to provide an experimental basis and theoretical guidance for improving the biological value of *S. nudus* L. by analyzing its immunomodulatory role for potential application as a functional food or prophylactic drug.

## 2 MATERIALS AND METHODS

### 2.1 Materials

*Sipunculus nudus* L. was purchased from the local Dongfeng Market in Zhanjiang, China. The entrails were removed immediately at the market. The body wall and meat were stored at −40°C until further use. Animal protease (enzyme activity 23 × 10^4^ U/g) and flavor proteases (enzyme activity 18 × 10^4^ U/g) were purchased from Pangbo Biological Engineering Co., Ltd. RPMI 1640 medium, DMEM medium, and penicillin–streptomycin solution were purchased from Gibco Life Technologies. Lipopolysaccharide (LPS) was procured from Sigma‐Aldrich. ConA was obtained from Yuanye Biotechnology. Fetal bovine serum was ordered from Gemini Bio‐Products. Neutral red was supplied by Tianxin Fine Chemicals. Cell Counting Kit‐8 was obtained from Dojindo Laboratories. The Griess Reagent System was ordered from Promega Corporation. Test kits of TNF‐α, IL‐6, and IL‐1β were supplied by ExCell Biotech. RAW 264.7 cells were derived from The Cell Bank of Type Culture Collection of the Chinese Academy of Sciences. RNA extraction kit, RNA reverse transcription kit, and qRT‐PCR kit were ordered from Vazyme Biotech Co., Ltd. All of the chemicals and reagents were of analytical grade. SPF Kunming mice (20 ± 2 g weight) were obtained from HFK Bioscience. All animal experiments were approved by the Animal Ethics Committee of Guangdong Ocean University (GDOU‐LAE‐2019‐0020). Primers for iNOS, TNF‐α, IL‐1β, IL‐6, and GAPDH genes were synthesized at Sangon Biotech, and GAPDH was used as an internal reference.

### 2.2 Preparation of enzymatic hydrolysates from *S. nudus* L.

*Sipunculus nudus* L. protein was prepared by alkali extraction and acid precipitation. The alkali extraction conditions were as follows: extraction temperature, 4°C; distilled water/solid ratio, 3:1 (v/w); pH, 12.5; and extraction time, 3 h. The pH of the extract was adjusted to 4.5, which was the isoelectric point of *S. nudus* L. protein, to obtain the flocculent precipitate. The precipitates were washed until the supernatant was colorless after centrifugation (10,000 rpm, 15 min), and the final protein products were obtained. Enzymatic hydrolysis was performed using animal protease and flavor proteases, and the enzyme‐to‐substrate (E/S) ratio was 2460 U/g. *Sipunculus nudus* L. protein was diluted by one‐fifth by redissolving in five times the volume of water (v/w), and the pH value was adjusted to 7.0. The protease was added to the protein solution, which was preheated in a constant temperature shaker at 56°C for 5 min, and the hydrolysis reaction lasted for 4.5 h. The hydrolysate was heated to 100°C for inactivation, followed by centrifugation for 20 min at 6500 r/min (TDL‐5‐A). The supernatant was desalted with a 100 Da reverse osmosis membrane, and the solution was filtered through a 2.5 kDa MWCO membrane and vacuum‐concentrated. After lyophilization, the enzymatic hydrolysate obtained from *S. nudus* L. was stored at −20°C until further use.

The content of *S. nudus* L. protein was determined via Kjeldahl method. The amino acid nitrogen was determined via formaldehyde titration. The degree of hydrolysis (DH) was calculated using the following formula: DH (%) = (hydrolyzed peptide bonds/total peptide bonds) × 100 = [(*B* − *C*)/(*A* − *D*)] × 100, where *A* is the total nitrogen in *S. nudus* L. protein; *B* denotes the amino acid nitrogen in *S. nudus* L. hydrolysate; and *C* and *D* represent the free‐formed amino acid nitrogen and nonprotein nitrogen in *S. nudus* L. protein, respectively.

### 2.3 Molecular weight distribution of peptides in enzymatic hydrolysate

The molecular distribution of samples was determined by high‐performance size‐exclusion chromatography. The samples of enzymatic hydrolysate of *S. nudus* L. were dissolved in distilled water and separated using an LC‐20 AD HPLC system equipped with a Waters Protein‐pak 60A (WAT 085250) column (column temperature of 25°C). The peptides were eluted with 0.05 mol/L Tris–HCl (pH 8.3) at a flow rate of 0.7 mL/min, and monitored at 214 nm. A calibration curve was obtained using the following standards (Sigma Chemicals): lysozyme (14,300 Da), bovine insulin (5733.49 Da), angiotensin II (1046.18 Da), *N*‐Hippuryl‐His‐Leu hydrate (429.47 Da), and tyrosine (181.19 Da). The regression equation was obtained using retention time (*t*) and molecular weight logarithm (LgM) as the axes: LgM = −0.1751 *t* + 5.4338 (*R* ^2^ = 0.9717).

### 2.4 Purification and identification of immunoregulatory peptide

The peptides (20.0 mg/mL) were eluted using ultrapure water in the column (2.6 × 65 cm) at a flow rate of 1.0 mL/min in the protein purification system (AKTA Purifier). All eluted fractions were monitored at 214 nm. Each fraction was pooled and vacuum freeze‐dried for determination of immunoregulatory activity. The peptide fraction with the highest immunoregulatory activity obtained with Sephadex G‐25 gel was separated and purified using acetonitrile and water (5:95, v/v) using a Symmetry C18 column (5 μm, 4.6 × 250 mm, Waters). All steps were performed at a flow rate of 1.0 mL/min. The immunoregulatory activity of all the fractions was determined. The peptide fraction with the highest immunoregulatory activity was separated by symmetry C18 column again with eluent A (ultrapure water), and then with a linear gradient of acetonitrile (5%–30%) containing 0.005% trifluoroacetic acid (TFA) at a flow rate of 1.0 mL/min for 15 min. All the fractions were collected and lyophilized and passed through a 0.20‐μm polytetrafluoroethylene (PTFE) syringe filter (Millex®) before determining its activity.

### 2.5 In vitro murine splenocyte proliferation assay

Splenocyte isolation and murine splenocyte proliferation assays were carried out as described previously (Li et al., 2019).

### 2.6 In vitro murine macrophage assay

Peritoneal macrophages were isolated from the peritoneal fluid. Cell preparation was performed as described before (Petricevich & Lebrun, 2005). Primary cells were cultured in DMEM medium supplemented with 10% FBS. Cells were grown at 37°C in a humidified 5% CO_2_ atmosphere. The cell suspension was diluted to 3.0 × 10^5^–5.0 × 10^5^ cells/mL. The phagocytosis of peritoneal macrophages was determined as described previously (Li et al., 2019). The NO content was analyzed using the Griess Reagent System G2930 (Promega Corporation) according to the manual instructions.

### 2.7 Amino acid sequences of purified peptide

The peptides separated by HPLC were then analyzed using tandem mass spectrometer (Q Exactive mass spectrometer, Thermo Fisher) for peptide identification under the following parameters: (1) peptide mass: resolution, 70,000; AGC target, 3 × 10^6^; maximum IT, 40 ms; scan range, 50–1800 m/z; (2) fragment mass: resolution, 17,500; AGC target, 1 × 10^5^; maximum IT, 60 ms; TopN, 20; NCE/stepped NCE, 27. The data obtained from the tandem mass spectrometer were searched in a database using Mascot software.

### 2.8 Characteristics and activity prediction of SNCP

The sequence alignment of related proteins was performed using T‐coffee tool (http://tcoffee.crg.cat/). The solubility of the peptide was evaluated using the online innovagen server, available at http://www.innovagen.com/proteomics‐tools. Basic biochemical properties were calculated with the ProtParam tool (https://web.expasy.org/protparam/). The toxicity of the peptide was predicted using ToxinPred (http://crdd.osdd.net/raghava/toxinpred/multisubmit.php).

The amino acid sequence of peptide was submitted to PeptideRanker (http://distilldeep.ucd.ie/PeptideRanker/) to determine the theoretical bioactivity of peptides, and the results were presented as score values ranging from 0 (poorest bioactivity) to 1 (best bioactivity) (Guo et al., 2020). Basic biochemical properties of the peptide were generated with a ProtParam tool (Gasteiger et al., 2005). Cell‐penetrating ability was assessed using four different online web servers (Kumar et al., 2018; Manavalan et al., 2018; Wei, Tang & Zou, 2017, Wei, Xing, et al., 2017). PreAIP method (http://kurata14.bio.kyutech.ac.jp/PreAIP/index.php) was used to predict the anti‐inflammatory activity.

### 2.9 Peptide synthesis

The peptides identified by LC–MS/MS were synthesized via solid‐phase peptide synthesis (China Peptides Co., Ltd.). The peptide synthesis entailed the following steps mainly: First, the C‐terminal carboxyl of the first amino acid was attached to the Wang resin, and then the protective group of N‐terminal amino was removed. Second, the amino acids were successively attached to the resin as described previously. Finally, the peptide was released from the resin and all protective groups were removed. The crude product was precipitated with cold ether and centrifuged at 5000 g for 10 min. The precipitate was dissolved in water or water/acetonitrile solution, and lyophilized to obtain a synthetic peptide. The purity of synthetic peptide was greater than 98% based on HPLC analysis (Figure S1). The amino acid sequence was identified by LC–MS/MS as RVKGKILAKRLN (Figure S2), which was consistent with the amino acid sequence of SNLP.

### 2.10 RAW 264.7 cell immunomodulatory activity

#### 2.10.1 Cell culture

RAW 264.7 cells were cultured in DMEM medium supplemented with 10% FBS, 1% of penicillin, and 1% of streptomycin in a humidified 5% CO_2_ atmosphere at 37°C. Cell concentration was adjusted to 3.0 × 10^5^–5.0 × 10^5^ cells/mL in order to increase the cell survival rate above 95%.

#### 2.10.2 Macrophage proliferation and phagocytosis

Cell proliferation was determined using the Cell Counting Kit according to the manufacturer's instructions by plating 3.0 × 10^5^–5.0 × 10^5^ cells /mL into 96‐well plates, followed by incubation overnight and treatment with different concentrations of the sample. The SNLP was passed through a 0.20‐μm PTFE syringe filter (Millex®) to avoid the interference of endotoxin or LPS (1 μg/mL) for 24 h. The supernatants were removed and the CCK8 (10 μL) solution was added, followed by incubation under the same conditions for 24 h. The absorbance at 450 nm was read on a microplate reader.

In addition, the cells in 96‐well plates (24 h) were washed three times with phosphate‐buffered saline (PBS), followed by the addition of 0.75 mL/L neutral red physiological saline solution (20 μL/well) and then cultured for another 1 h. Finally, the cells were washed with PBS again, and glacial acetic acid–ethanol (1:1, v/v) lysis solution was added (100 μL/well). The plates were transferred to room temperature for 30 min. Absorbance was measured at 540 nm with the microplate reader.

#### 2.10.3 Assay of phagocytosis and cytokine secretion

Cells were cultured for 24 h. The supernatant was collected, and the NO, TNF‐a, IL‐6, and IL‐1β levels were assessed by Griess Reagent System G2930 (Promega Corporation) and the ELISA kit (Mouse TNF‐α, IL‐6, and IL‐1β, ExCell Bio), according to the manufacturer's protocol.

#### 2.10.4 Real‐time quantitative polymerase chain reaction (qRT‐PCR)

Following treatment with the sample and LPS for 24 h, the total RNA in RAW 264.7 cells was isolated and reverse‐transcribed to cDNA. The iNOS, TNF‐α, IL‐1β, IL‐6, and GAPDH cDNA were amplified via PCR using Premix Taq (Vazyme) under the following parameters: 1 cycle for 30 s at 95°C; 40 cycles for 3–10 s at 95°C, 10–30 s at 60°C, and 15 s at 95°C; and 1 cycle for 60 s at 60°C, 15 s at 95°C. The primer sequences are listed in Table 1S.

### 2.11 Statistical analysis

The data are presented as the means ± standard deviation. They were analyzed using one‐way analysis of variance (ANOVA) to determine any significant differences with GraphPad Prism 7 (GraphPad Software, Inc.). A value of *p* < .05 was considered significant. Compared with the control group, * means significant (*p* < .05), and ** denotes extremely significant differences statistically (*p* < .01).

## 3 RESULTS

### 3.1 Properties and immunoregulatory activities of enzymatic hydrolysate

**FIGURE 1:**
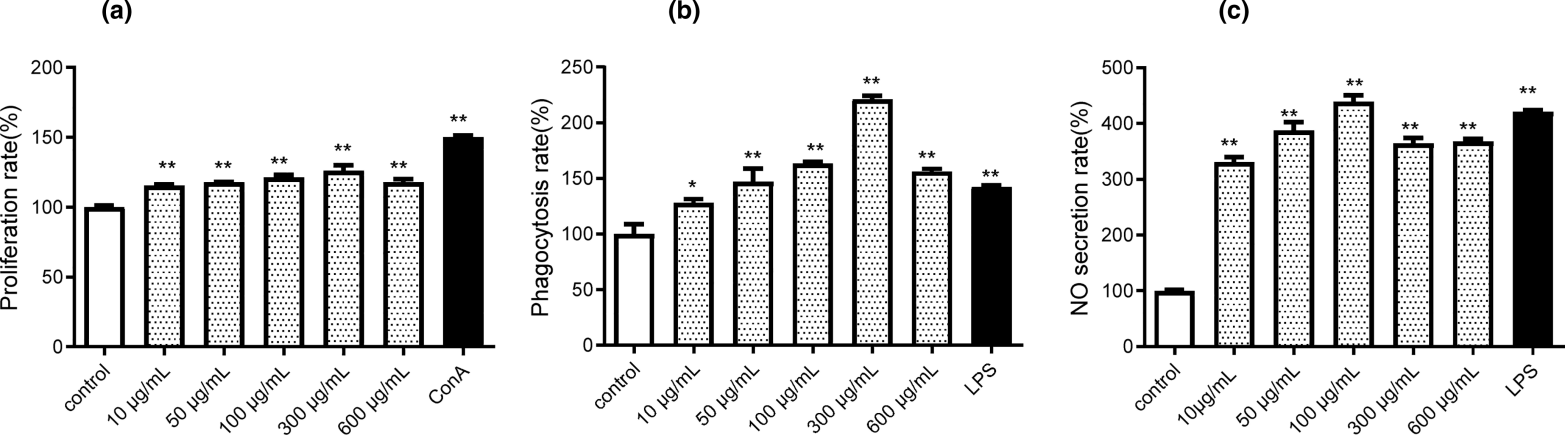
Evaluation of the immunoregulatory activity of enzymatic hydrolysate of *Sipunculus nudus* L. in vitro (*n* = 3). (a) The proliferation rates of spleen lymphocytes; (b) The phagocytosis of neutral red by peritoneal macrophages; (c) The phagocytosis of NO production by peritoneal macrophages. Compared with control group, *p < .05, **p < .01.

To characterize the properties and immunoregulatory activities of the *S. nudus* L. enzymatic hydrolysate, we analyzed the degree of hydrolysis and molecular weight distribution as well as the capacity to stimulate immune cell proliferation and phagocytosis, and NO production. Isoelectric focusing was used to isolate protein and to exclude other components such as polysaccharides and fatty acids, followed by enzymatic hydrolysis and ultrafiltration. Formaldehyde titration and gel filtration chromatography were used to determine the degree of hydrolysis and molecular weight distribution of the hydrolysate, respectively. Results showed that the degree of hydrolysis was 38.92%, which suggested a highly efficient enzymatic hydrolysis. The results of size‐exclusion chromatography showed that 95.62% of the peptides in the hydrolysate were smaller than 2.5 kDa (Table 1), which suggests that the hydrolysate is mainly a mixture of small peptides. As shown in Figure 1, the hydrolysate increased the proliferative level of spleen lymphocytes as well as the phagocytosis of the macrophages (Figure 1a,b). In addition, the hydrolysate significantly raised the NO levels in macrophages (Figure 1c). In conclusion, the hydrolysate, mainly composed of small peptides, shows significant immunoregulatory activities and thus is a promising source of immunoregulatory peptides.

**TABLE 1:** Molecular weight distribution of the enzymatic hydrolysate of *Sipunculus nudus* L.

Molecular weight	<2.5 kDa	>2.5 kDa
Percentage (%)	95.62	4.38

### 3.2 Purification and identification of immunoregulatory peptide

**FIGURE 2:**
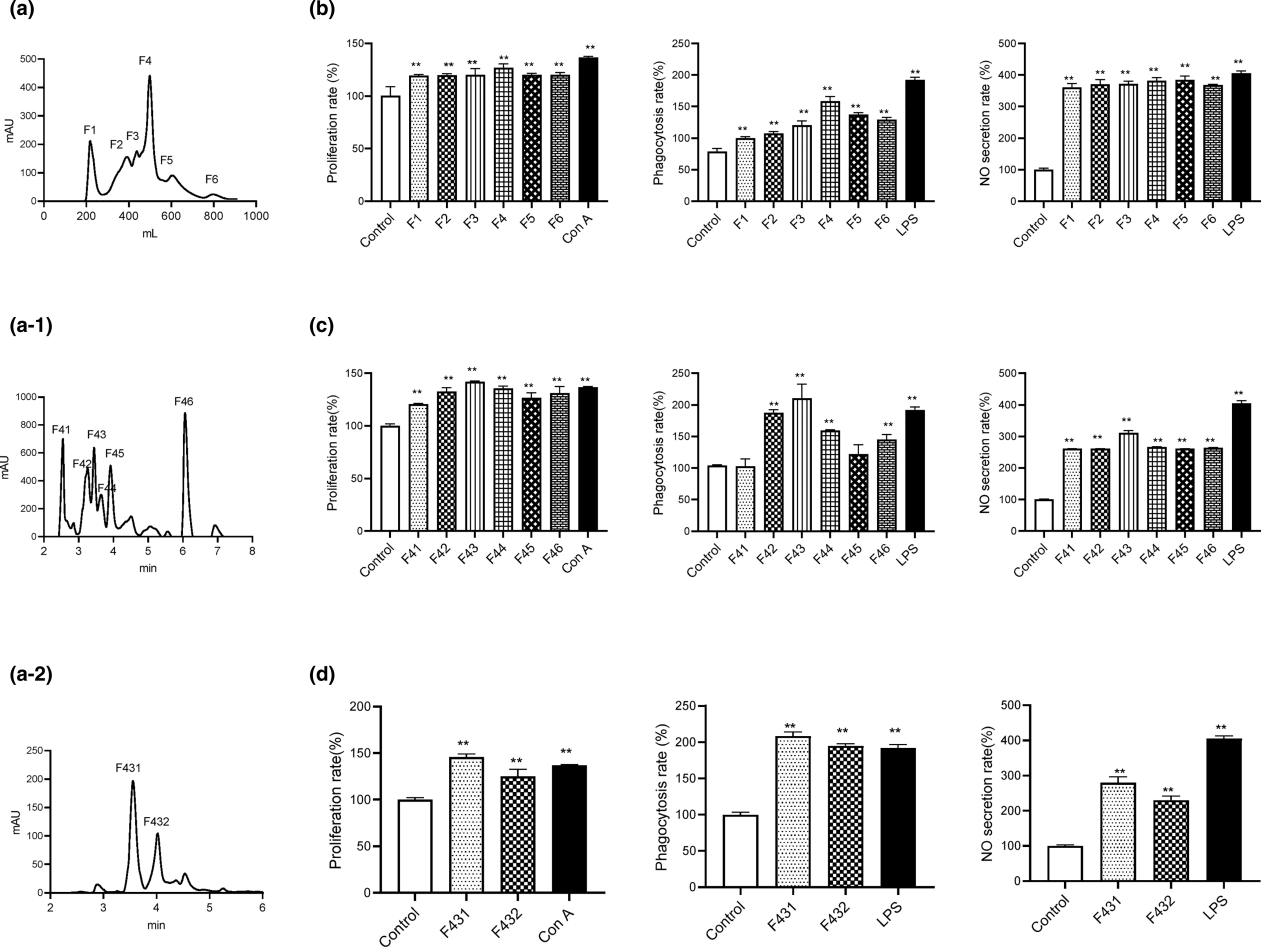
Elution curves of the fractions separated from enzymatic hydrolysate of *Sipunculus nudus* L. and the effects on immunoregulatory activity. (a, b) The elution curve of the peptides via Sephadex G‐25 and the immunoregulatory activities of the elution components (F1 ~ F6). (a‐1, c) RP‐HPLC chromatography of F4 and the immunoregulatory activities of the elution components. (a‐2, d) RP‐HPLC chromatography of F43 and the immunoregulatory activities of F431 and F432.(b–d) The subgraphs from left to right are the proliferation rates of spleen lymphocytes, the phagocytosis of neutral red and the NO production by peritoneal macrophages, respectively (*n* = 3). Compared with control group, *p < .05, **p < .01.

**FIGURE 3:**
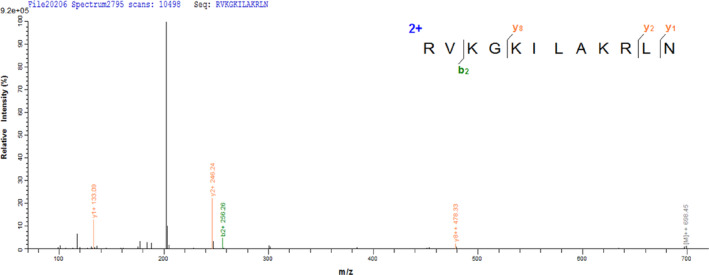
MS/MS spectra of SNLP identified and screened from the F431 fraction.

The aforementioned hydrolysate consists of a mixture of peptides and the immunoregulatory activities are contributed by one or more peptides in the hydrolysate. To purify and identify the peptide, gel filtration (Sephadex G‐25 column) and RP‐HPLC (Symmetry C18 column) were utilized, and the levels of lymphocyte proliferation, phagocytosis, and NO production of murine macrophages were used to screen the fractions. As shown in Figure 2a, six different fractions (F1, F2, F3, F4, F5, and F6) were collected and all the fractions significantly increased the levels of lymphocyte proliferation and phagocytosis compared with the control (*p* < .05). F4 fraction showed the most potent immunoregulatory activities and thus was analyzed via RP‐HPLC. The first RP‐HPLC generated six fractions, and the fraction with the strongest activity (F43) was further analyzed via a second RP‐HPLC (Figure 2b). Two fractions (F431 and F432) were obtained, and the F431 fraction showed stronger immunoregulatory activities (Figure 2c). Therefore, the amino acid sequence of F431 was determined by LC–MS/MS as Arg–Val–Lys–Gly–Lys–Ile–Leu–Ala–Lys–Arg–Leu–Asn (RVKGKILAKRLN, designated as SNLP) (Figure 3).

### 3.3 Characteristics and activity prediction of SNCP

#### 3.3.1 SNLP originates from the putative ribosomal protein L21 in *S. nudus* L.

The functions of the parent protein usually provide useful peptide functional information. Hence, we searched for the parent protein of the SNLP and investigated its ligand binding sites. Proteins sharing strong identity with SNLP in PSI‐BLAST results were largely derived from ribosomal protein L21. Results showed that SNLP originated from the putative ribosomal protein L21 in *S. nudus* (L21‐SN), which was highly homologous to ribosomal protein L21 obtained from *Penaeus vannamei*, *Callorhinchus milii*, and *Tupaia chinensis* (Figure S3).

#### 3.3.2 SNLP is a novel nontoxic bioactive peptide with good water solubility

SNLP released from *S. nudus* L. protein was further analyzed to screen for novel bioactive peptides with specific properties. PeptideRanker was used to predict the theoretical bioactivity of peptide with a core value of 0.2422, with no previous bioactivity based on BIOPEP database and studies.

As shown in Table 2, the prediction of biochemical properties showed that SNLP was nontoxic and soluble in water due to the presence of strongly hydrophilic residues (hydropathicity of −0.542). SNLP is positively charged (+5) at pH 7 based on calculations, and the instability index of 28.76 indicates that SNLP is a stable peptide (Table 2).

**TABLE 2:** Characteristics of SNLP.

Peptide	Protein	PeptideRanker score	Toxicity	Solubility	Molecule W	theoretical pI	Net charge at pH 7	Instability index
RVKGKILAKRLN	putative ribosomal protein L21	0.2423	Nontoxin	Good water solubility	1395.74 g/mol	12.18	5	28.76

#### 3.3.3 SNLP is a probable cell‐penetrating peptide (CPP) and anti‐inflammatory peptide (AIP)

Positive charge is an important characteristic of cell‐penetrating peptides (CPPs), which cross the cell membrane without interacting with any specific receptors on the membrane (Ramsey & Flynn, 2015). Predictions of CPPs based on machine learning, sequencing, and 3‐D structural analyses in recent years have been highly accurate (Zhang et al., 2020). Therefore, four online servers (Kumar et al., 2018; Manavalan et al., 2018; Wei, Tang & Zou, 2017, Wei, Xing et al., 2017) were used to predict whether or not the positively charged SNLP was CPP. As shown in Table 3, the peptide is a cell‐penetrating peptide with efficient uptake.

**TABLE 3:** Predicting the probabilities of SNLP as cell‐penetrating peptide (CPP) and anti‐inflammatory peptide (AIP).

Number	Type	Probability	Uptake efficiency	Probability score	References
1	CPP	0.857	Low	0.350	Manavalan et al. (2018)
2	CPP	0.790	High	0.570	Wei, Tang, and Zou (2017), Wei, Xing, et al. (2017)
3	CPP	0.287	N/A	N/A	Kumar et al. (2018)
4	CPP	0.953	N/A	N/A	Wei, Tang & Zou (2017a)
5	AIP	0.574	N/A	N/A	Khatun et al. (2019)

**FIGURE 4:**
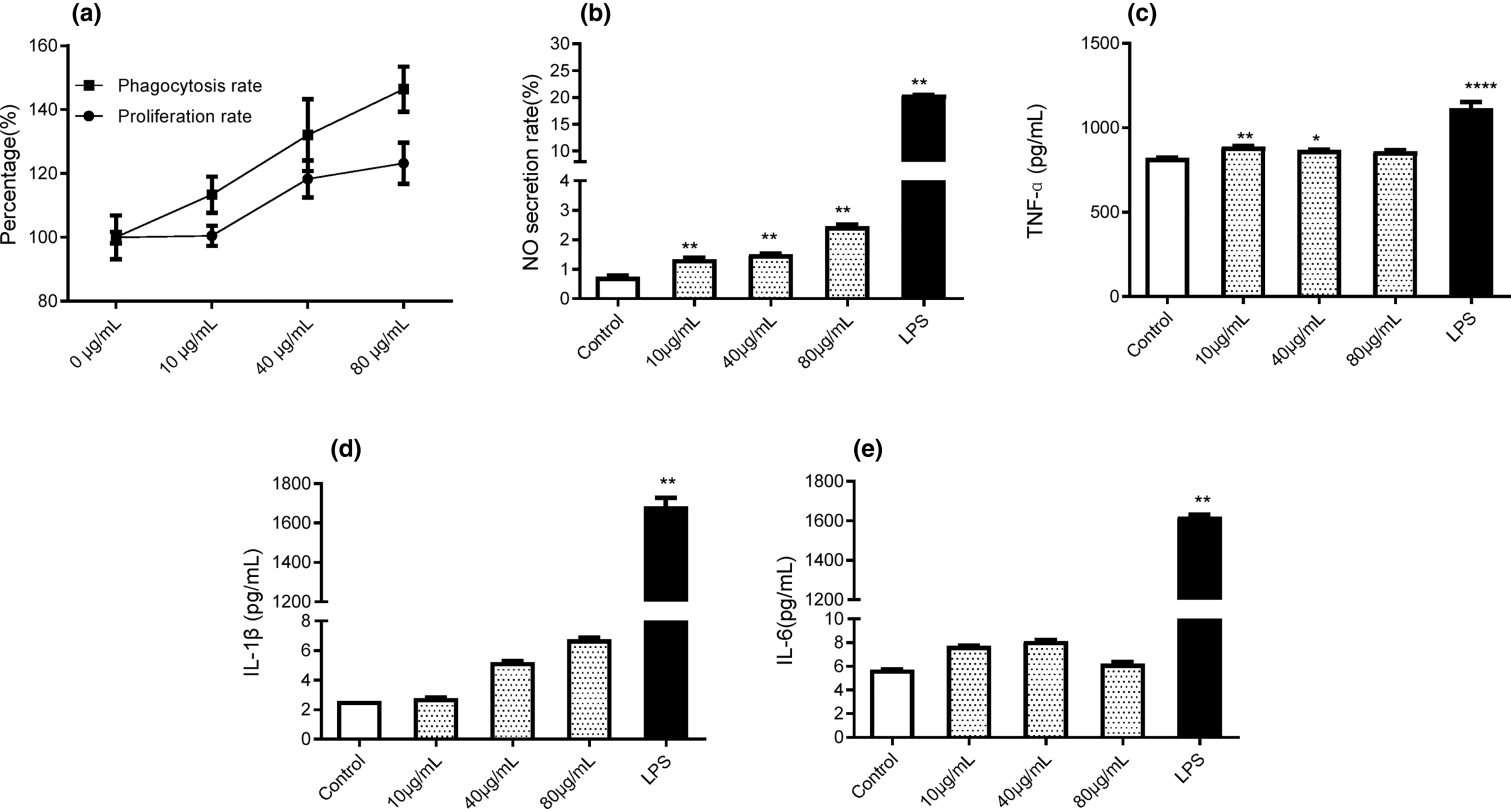
Evaluation of the immunoregulatory activity of synthetic SNLP in vitro (*n* = 3). (a) Effects of SNCP on the proliferation and the phagocytosis rates of RAW 264.7 cells. (b–e) The cytokines (NO, TNF‐ɑ, IL‐1β, and IL‐6, respectively) production after treatment by the synthetic SNLP.

Inflammation in the cell triggers the production of cytokines via immune response following exposure to bacterial toxins and other pathogens; however, overwhelming inflammation in the body is detrimental to cells (Gupta et al., 2018). Numerous anti‐inflammatory peptides (AIPs) have, therefore, been considered as candidate anti‐inflammatory drugs for the treatment of autoimmune and inflammatory illnesses. To determine whether SNLP is an AIP, the PreAIP method (http://kurata14.bio.kyutech.ac.jp/PreAIP/index.php) was used in this study to analyze both the primary sequence and structural properties (Khatun et al., 2019). The results suggest that SNLP is an anti‐inflammatory peptide with a high confidence score of 0.574 (Table 3). This is consistent with the two‐fold beneficial effects of SNLP including stimulation of lymphocyte proliferation without damaging the immune cells (Figure 4).

### 3.4 Immunoregulatory activity of the synthesized SNLP

To avoid possible interference due to unexpected impurities in SNLP, we synthesized SNLP to verify its immunoregulatory activity. RAW 264.7 cells are a widely used macrophage cell line to measure the immunoregulatory activity of bioactive substances. Therefore, we tested the abilities of synthetic SNLP to stimulate the proliferation, phagocytosis, and secretion of NO and cytokines (TNF‐α, IL‐1β, and IL‐6) in RAW 264.7 cells. The cells were treated with 10, 40, and 80 μg/mL of synthetic SNLP. As shown in Figure 4a, synthetic SNLP increases the proliferation level of macrophages in a concentration‐dependent manner (10.0–80.0 μg/mL), which indicates that SNLP is nontoxic to macrophages. Further, SNLP significantly increased the cellular uptake of neutral red dye (*p* < .05, vs. control group) in a dose‐dependent manner, which indicated that SNLP had the ability to enhance phagocytosis of RAW264.7 cells. SNLP exerted a significant stimulatory effect on NO production in a concentration‐dependent pattern, even though the NO release by cells was less than in the LPS group (Figure 4b). These results suggested that SNLP treatment mediated the upregulation of NO secretion. The levels of TNF‐α, IL‐1β, and IL‐6 in RAW264.7 cells were increased significantly by SNLP (*p* < .05) (Figure 4b–e). Compared with the control group, SNLP significantly promoted the secretion of TNF‐α and IL‐1β levels in RAW 264.7 cells in a concentration‐dependent manner (Figure 4b–d). However, no significant differences in IL‐6 levels compared with the control group were found (Figure 4e). These results are consistent with the prediction that SNLP was an AIP. The addition of LPS to the cells induced substantial levels of these cytokines, which were significantly higher than in the SNLP groups (*p* < .05). These results indicated that SNLP strengthened the immune function of RAW264.7 cells by boosting the secretion of IL‐6, TNF‐α, and IL‐10.

**FIGURE 5:**
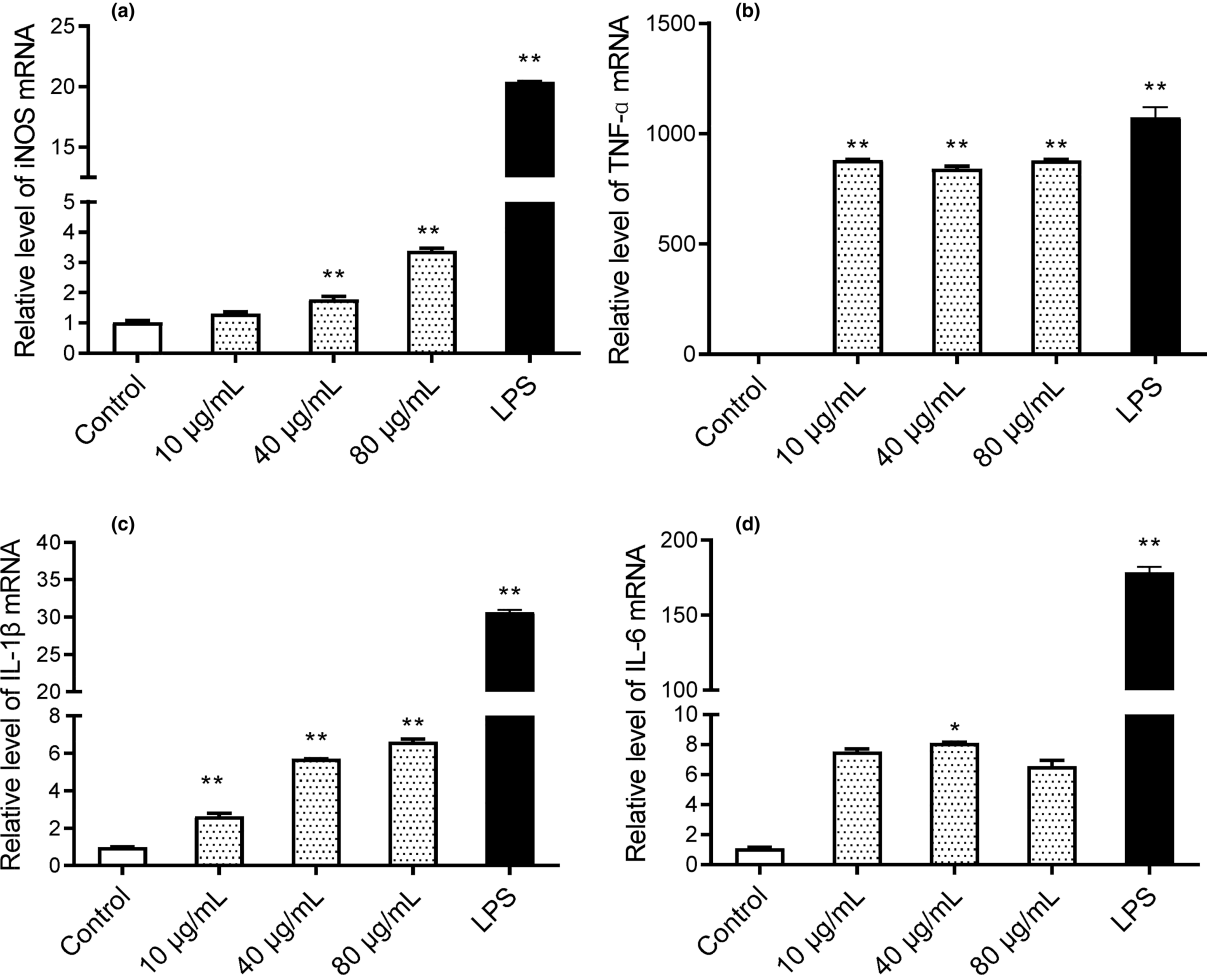
Effects of SNLP on the mRNA expression of cytokines in RAW264.7 (NO, TNF‐ɑ, IL‐1β, and IL‐6) (*n* = 3). Compared with control group, *p < .05, **p < .01.

To further confirm the effect of SNLP on NO and cytokine synthesis, the mRNA levels of iNOS, IL‐1β, IL‐6, and TNF‐α in RAW 264.7 cells were investigated by qRT‐PCR. As shown in Figure 5, compared with the LPS group, the mRNA levels of iNOS and cytokines (IL‐1β, TNF‐α, and IL‐6) were downregulated when treated with three different doses of SNLP.

In conclusion, synthetic SNLP exhibits potent immunoregulatory activity by increasing the proliferation and phagocytosis of RAW 264.7 cells and regulating the production of cytokines and NO.

## 4 DISCUSSION

*Sipunculus nudus* L. contains a large proportion of protein component, which has been neglected in recent years. Our pilot study indicates potent immunoregulatory effects of the enzymatic hydrolysate derived from *S. nudus* L. Our goal in this study was to isolate and identify immunoregulatory peptides with potent activities. A combination of techniques, including protein isolation, enzymatic hydrolysis, ultrafiltration, size‐exclusion chromatography, and RP‐HPLC, were utilized for purification. A novel peptide SNLP was purified and identified as RVKGKILAKRLN. SNLP originates from the putative ribosomal protein L21 in *S. nudus* L. It is a novel bioactive peptide without toxic side effects and exhibits good water solubility. It is a probable AIP with cell‐penetrating ability. The immunoregulatory activity of the synthesized SNLP was verified using RAW 264.7 cells in vitro. SNLP showed potent activities in promoting proliferation and phagocytosis of macrophages, and regulating the production of cytokines and NO.

### 4.1 Enzymatic hydrolysate of *S. nudus* L. as a source of immunoregulatory peptides

Enzymatic hydrolysis, microbial fermentation, and heat treatment are the most common methods utilized to generate peptides from edible proteins. Enzymatic hydrolysis has been preferred in recent years due to its safety, mild reaction conditions, and easy‐to‐control process (Chalamaiah et al., 2015). A number of biological peptides were produced using enzymatic techniques including antioxidant peptides (Agrawal et al., 2019; Hu, Pan et al., 2022, Hu, Yang et al., 2022), angiotensin converting enzyme (ACE) inhibitory peptides (Sangiorgio et al., 2022; Xie et al., 2022), and immunoregulatory peptides (Lee & Paik, 2019; Yu et al., 2020). These peptides were derived from animal and plant hydrolysates, which generally contain a large number of peptides that are smaller than 5 kDa. Further, multiple studies suggest that lower molecular weight peptides were more effective immunomodulators, and induced macrophage proliferation, secretion of cytokines, and inflammatory mediators (He et al., 2021). Xu et al. (2020) collected six fractions of enzymatic hydrolysate and the fraction (smaller than 1 kDa) exhibited the most potent macrophage proliferation (Xu et al., 2020). Zhang et al. (2021) isolated and purified selenium‐enriched soybean peptides (<3 kDa, Se‐SPep) from the selenium‐enriched soybean protein (Se‐SPro), which exhibited immunoregulatory activity (Zhang et al., 2021). The hydrolysate obtained in our study consisted of largely small peptides (<2.5 kDa, which accounted for 95.62%) (Table 1). It facilitated immune cell proliferation and phagocytosis, as well as NO production (Figure 1).

### 4.2 The peptide, SNLP, is a potent immunoregulatory peptide

The combination of size‐exclusion chromatography and RP‐HPLC is a common and effective way to separate and purify bioactive peptides (Waili et al., 2021). In this study, the Sephadex G‐25 column was used to separate the relative complex hydrolysate based on the size of the peptides. RP‐HPLC was used to purify the peptide due to its high resolution in recognizing similar peptides. Finally, the most potent peptide (F431) was obtained, and the sequence was identified as RVKGKILAKRLN by LC–MS/MS (Figure 3).

The length, hydrophobicity, and charges are closely related to the biological function of peptides. Shorter peptide is more resistant to enzyme digestion and is easily absorbed by intestines and thus it exerts potent biological activities (Roberts et al., 1999). The biological peptides identified before generally range from 2 to 20 amino acids (Xu et al., 2020). It has been reported that hydrophobicity is needed for various biological functions such as antioxidant, ACE inhibitory, and antimicrobial activity. This is attributed to the helical structures of the peptide forming perfect amphipathic conformations by separating hydrophobic and hydrophilic amino acids under different planes (Pasupuleti et al., 2012) and thereby increasing the interaction between the amphipathic surface and other potential targets. However, higher hydrophobicity increases the cytotoxic effects of peptides due to insolubility in aqueous environment, and thus the peptide is more likely to interrupt the membrane integrity (Pasupuleti et al., 2012). Cationic charge of the peptide is attributed to the presence of positively charged amino acids such as arginine and lysine. Membrane contains negatively charged moieties, protein receptors, and other anionic molecules (Meloni et al., 2020). This suggests that the cationic peptide is biologically active against multiple targets. In general, SNLP, with a small molecular and positive charge, is a potential active peptide.

SNLP strongly increased immune cell proliferation and phagocytosis as well as NO production (Figure 2). Lymphocytes including T cells, B cells, and natural killer cells regulate both innate and adaptive immunity, and lymphocyte proliferation is considered as a common immunoregulatory mechanism (Gill et al., 2000). Macrophages recognize a large number of potentially harmful particles such as pathogens and initiate phagocytosis to eliminate them (Chalamaiah et al., 2018). Immunoregulatory peptides derived from milk, whey, egg, soy, and pine nut have been shown to induce the proliferation of immune cells and phagocytosis (Santiago‐López et al., 2016; Sun et al., 2020).

### 4.3 Potential immunomodulatory mechanisms of SNLP

Macrophages, the most crucial sentinels of the innate immune system, contribute to host defense and resistance to bacteria, toxins, or other pathogens or invaders (Khatun et al., 2019). Therefore, the exogenous activation of macrophages is an effective strategy to upregulate the innate immune response (He et al., 2021). We first evaluated the cytotoxicity, proliferation, and phagocytosis of SNLP. Our results showed that SNLP, under the test concentrations (10.0–80.0 μg/mL), was nontoxic against RAW264.7 cells. SNLP induced the proliferation and phagocytosis of RAW264.7 cells.

Cytokines and inflammatory mediators were also secreted by activated macrophages during the host defense response. It has been reported that inflammation is part of the immune system's response to disease or tissue damage. NO has been reported as a crucial mediator of inflammatory response to invading pathogens, eliminating tumor cells, and performing biological functions. The activated macrophages secrete pro‐inflammatory cytokines, such as IL‐6, IL‐1β, and TNF‐α to defend against the pathogens (He et al., 2021; Hossen et al., 2023). However, excessive production of NO and pro‐inflammatory cytokines can lead to increased tissue damage and induce serious immune dysfunction in the body. Therefore, a moderate inflammatory immune response facilitates the elimination of pathogens and contributes to self‐repair (Ru et al., 2021). It has been reported that several immunomodulatory peptides exhibit both immunostimulatory and anti‐inflammatory activities (Ahmad et al., 2019; Yu et al., 2023).

In this study, the computer‐based prediction indicated that SNLP is a probable AIP with cell‐penetrating ability (Table 3). Therefore, we determined the levels of NO and pro‐inflammatory cytokines to determine whether SNLP was an AIP. Our results indicated that SNLP strengthened the immune function of RAW 264.7 cells by boosting the secretion of IL‐6, IL‐1β, and TNF‐α (Figure 4). Both TNF‐α and IL‐1β are pro‐inflammatory cytokines, while IL‐6 has both pro‐inflammatory and anti‐inflammatory functions (Kany et al., 2019; Rose‐John, 2018). Although these pro‐inflammatory cytokines are generally involved in inflammatory diseases, a number of immunoregulatory peptides upregulate the effects of pro‐inflammatory cytokines (Santiago‐López et al., 2016). Synthetic SNLP significantly induced IL‐1β expression without affecting the proliferation of immune cells (Figure 4). The expression levels of cytokines (IL‐6, TNF‐α, and IL‐1β) were significantly lower than the detrimental levels induced by LPS (Figure 5). Our results provide preliminary evidence suggesting that SNLP acts as a dual immunomodulatory peptide with both immunostimulatory and anti‐inflammatory activities.

Many factors affect protein–mRNA correlation such as delayed synthesis, availability of cellular resources, and other conditions (Liu et al., 2016). The inconsistency between the cytokine and mRNA levels induced by SNLP needs to be further investigated. The NO production is not only lethal to pathogens but also regulates downstream pathways in immune system such as cytokine induction and proliferation of immune cells (Bogdan, 2001). Following invasion by pathogens, the inducible nitric oxide synthase (iNOS) catalyzes the synthesis of NO from L‐arginine in macrophages, and indirectly activates lymphocytes and other immune cells, resulting in response to inflammation and prompt elimination of pathogens (Deng et al., 2023). As shown in Figures 4 and 5, the synthetic SNLP promotes the NO production but not to toxic levels observed in the LPS group. SNLP also increased the mRNA level of iNOS, which is responsible for NO synthesis. In addition, the macrophages produced NO regulated by cytokines (Bogdan, 2001). The results suggested that SNLP increased the proliferation of macrophages, which was consistent with the increased NO production.

In brief, SNLP stimulates the proliferation and phagocytosis of immune cells, increases the NO production and mRNA level of iNOS, and regulates the transcription of cytokines. Thus, SNLP is a potent immunoregulatory peptide with anti‐inflammatory activity.

## 5 CONCLUSIONS

An immunoregulatory peptide from *S. nudus* L. was purified and characterized via multiple techniques including enzymatic hydrolysis, ultrafiltration, gel filtration chromatography, tandem RP‐HPLC, and LC MS/MS in this study. The underlying immunoregulatory mechanisms have been investigated. The immunoregulatory activity was measured based on the lymphocyte proliferation rate and phagocytosis as well as the NO levels to screen the peptide fractions. Finally, a novel immunoregulatory peptide named as SNLP was identified as Arg–Val–Lys–Gly–Lys–Ile–Leu–Ala–Lys–Arg–Leu–Asn (RVKGKILAKRLN). The synthesized SNLP exhibited potent immunoregulatory activity. SNLP was found to increase the proliferation and phagocytic activity of RAW264.7 macrophages. It significantly promotes the cellular production of NO and cytokines such as TNF‐α, IL‐1β, and IL‐6. Our results provide preliminary evidence suggesting that SNLP is a dual immunomodulatory peptide with immunostimulatory and anti‐inflammatory activities. In conclusion, SNLP is a potent immunoregulatory peptide and represents a promising candidate for functional food or immunoregulatory drug.

## AUTHOR CONTRIBUTIONS

**Haisheng Lin:** Conceptualization (lead); data curation (equal); investigation (equal); methodology (equal); writing – original draft (equal). **Wan Li:** Data curation (equal); investigation (equal); methodology (equal); writing – original draft (equal). **Ruikun Sun:** Data curation (equal); methodology (equal); writing – review and editing (equal). **Cheng Xu:** Software (lead); writing – review and editing (equal). **Chaohua Zhang:** Supervision (lead); validation (lead). **Jialong Gao:** Writing – review and editing (equal). **Wenhong Cao:** Writing – review and editing (equal). **Xiaoming Qin:** Writing – review and editing (equal). **Saiyi Zhong:** Formal analysis (lead). **Yibin Chen:** Writing – review and editing (equal).

## FUNDING INFORMATION

This work was supported by the Innovative Team Program of High Education of Guangdong Province (2021KCXTD021), the Guangdong Higher Education Institution Innovative Team of High Value Processing and Utilization of Aquatic Products (GDOU2016030503), and Science and Technology Bureau of Zhanjiang (2019A01022). We are grateful to anonymous referees for their comments and constructive suggestions provided for improving the manuscript.

## CONFLICT OF INTEREST STATEMENT

The authors declare no conflict of interest.

## ETHICS STATEMENT

This study was approved by the Animal Ethics Committee of Guangdong Ocean University (approval number: GDOU‐LAE‐2019‐0020).

## Supporting information


Data S1:

## Data Availability

The data that support the findings of this study are available from the corresponding author upon reasonable request.
