# Supplementary material for: Purification and characterization of a novel immunoregulatory peptide from Sipunculus nudus L. protein
Source: Food Sci Nutr. 2023 Sep 26;11(12):7779–90. doi: 10.1002/fsn3.3695 (PMC10724601; doi:10.1002/fsn3.3695)
Supplement: Supplementary file 1 — Data S1: [file FSN3-11-7779-s001.docx]

**Supplementary Materials**

**Supplemental Table 1S.** The primer sequences for cytokines, iNOS and GAPDH

| **Gene** | **Forward primer** | **Reverse primer** |
| --- | --- | --- |
| GAPDH | CAGGAGGCATTGCTGATGAT | GAAGGCTGGGGCTCATTT |
| iNOS | CCCTTCCGAAGTTTCTGGCAGCAG | GGCTGTCAGAGCCTCGTGGCTTTGG |
| TNF-α | GGTCAATCTGCCCAAGTA | CACCCATTCCCTTCACAG |
| IL-6 | TTGACCTCAGCGCTGAGTTG | CCTGTAGCCCACGTCGTAGC |
| IL-1β | TATGGGCTGGACTGTTTCTAATGC | TTCTTGTGACCCTGAGCGACCT |


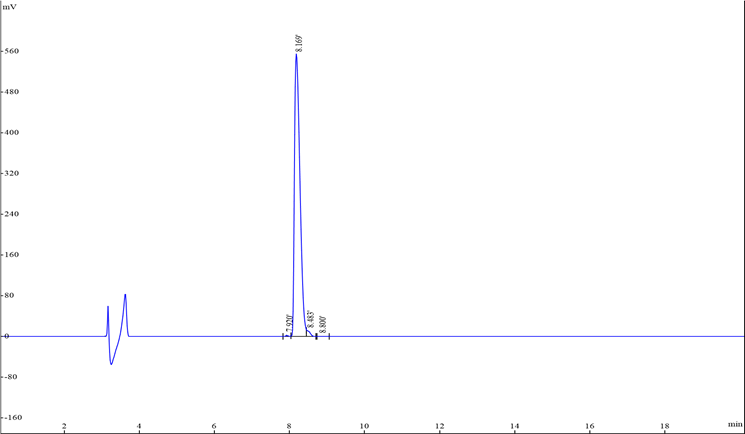


**Supplemental Figure 1S.** HPLC analysis of synthetic SNLP


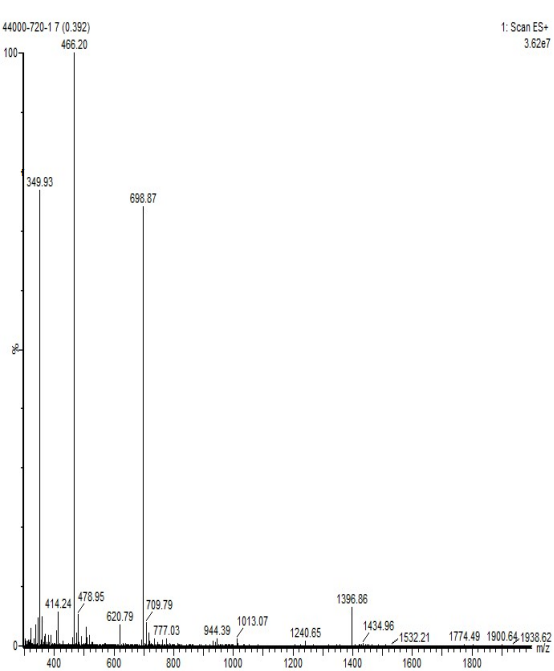


**Supplemental Figure 2S.** Peptide identification from synthetic SNLP via LC-MS/MS. The amino acid sequence was identified as RVKGKILAKRLN.


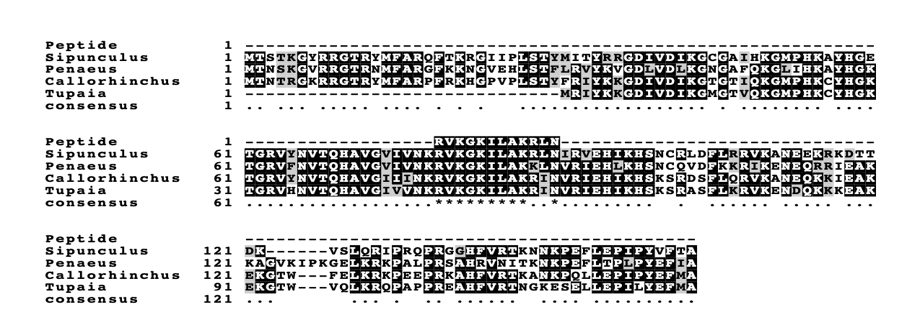
**.**

**Supplemental Figure 3S.** The sequence alignment between SNLP and the Ribosomal Protein L21 of *Sipunculus nudus, Penaeusvannamei, Callorhinchusmilii, Tupaiachinensis*.
